# Gut microbiota analyses of cutaneous T-cell lymphoma patients undergoing narrowband ultraviolet B therapy reveal alterations associated with disease treatment

**DOI:** 10.3389/fimmu.2023.1280205

**Published:** 2024-01-11

**Authors:** William Q. Nguyen, Lauren P. Chrisman, Gail L. Enriquez, Madeline J. Hooper, Teresa L. Griffin, Merjaan Ahmad, Sophia Rahman, Stefan J. Green, Patrick C. Seed, Joan Guitart, Michael B. Burns, Xiaolong A. Zhou

**Affiliations:** ^1^ Department of Dermatology, Northwestern University, Feinberg School of Medicine, Chicago, IL, United States; ^2^ Department of Biology, Loyola University Chicago, Chicago, IL, United States; ^3^ Genomics and Microbiome Core Facility, Rush University Medical Center, Chicago, IL, United States; ^4^ Division of Pediatric Infectious Diseases, Ann & Robert H. Lurie Children’s Hospital of Chicago, Chicago, IL, United States; ^5^ Department of Pediatrics, Northwestern University, Feinberg School of Medicine, Chicago, IL, United States

**Keywords:** cutaneous T cell lymphoma, microbiome, phototherapy, gut dysbiosis, cancer, lymphoma, skin cancer, inositol

## Abstract

Recent studies have shown a close relationship between cutaneous T-cell lymphoma (CTCL) and its microbiome. CTCL disease progression is associated with gut dysbiosis and alterations in bacterial taxa parallel those observed in immunologically similar atopic dermatitis. Moreover, the microbial profile of lesional skin may predict response to narrowband ultraviolet B (nbUVB), a common skin-directed therapy. However, the relationship between the gut microbiome, an immunologically vital niche, and nbUVB remains unexplored in CTCL. Herein, we performed 16S rRNA sequencing and PICRUSt2 predictive metagenomics on DNA extracted from stool swabs of 13 CTCL patients treated with nbUVB, 8 non-treated patients, and 13 healthy controls. Disease response was assessed with modified Severity Weighted Assessment Tool (mSWAT); of nbUVB-treated patients, 6 improved (decreased mSWAT), 2 remained stable, and 5 worsened (increased mSWAT). Protective commensal bacteria including *Lactobacillaceae* and *Erysipelatoclostridiaceae* were significantly less abundant in CTCL patients compared to controls. With treatment, the CTCL gut microbiome exhibited decreased phylogenetic diversity and lower relative abundance of pro-inflammatory *Sutterellaceae*. *Sutterellaceae* was also significantly more abundant in patients who worsened, and *Eggerthellaceae* and *Erysipelotrichaceae* trended higher in patients who improved. Finally, PICRUSt2 functional predictions based on shifts in abundance of bacterial sequences repeatedly identified alterations in inositol degradation, which plays a key role in host immunomodulation, including inositol phospholipid signaling relevant to T-cell survival and proliferation. Our results bolster the paradigm of gut dysbiosis in CTCL and its functional implications in disease pathogenesis, and further delineate bacterial taxa associated with nbUVB response and with nbUVB treatment itself.

## Introduction

Cutaneous T-cell lymphoma (CTCL) encompasses a heterogeneous group of non-Hodgkin T-cell lymphomas that home to the skin (1). Patients with advanced CTCL suffer from immune dysregulation (2–5), increasing their susceptibility to infections and leading to sepsis, organ failure, and death (6, 7). Increasing evidence suggests that CTCL is closely tied to the host microbiome. Broad-spectrum antibiotic treatment correlates with reduced tumor burden in some patients, and spontaneous CTCL mouse models exhibit mild, indolent disease when housed in germ-free isolators but have rapid disease progression when moved to traditional housing (8, 9). Recent work published by our group and others has demonstrated that CTCL patients are globally dysbiotic, with altered skin, gut, and nasal microbiomes (10–16) and that dysbiosis worsens with disease severity (10–12, 16, 17).

Our prior work on the gut microbiome of CTCL patients revealed loss of bacteria known to be butyrate producers, gut epithelial protectors, and anti-inflammatory mediators (11). We also showed that gut microbial shifts in CTCL paralleled those observed in atopic dermatitis but opposed those seen in psoriasis and other chronic skin diseases with similar and differing immunologic profiles, respectively. While this bidirectional gut-skin axis is now an area of active study in many inflammatory skin diseases (18), very few studies have examined the impact of a skin/tumor-directed therapy on the gut microbiome.

We previously examined the skin microbiome of CTCL patients treated with standard of care narrow-band ultraviolet B (nbUVB) phototherapy (19), a type of skin-directed therapy, and demonstrated that 1) microbial diversity increased in responders but not in non-responders; 2) relative abundances of particular bacterial taxa (e.g. *Staphylococcus capitis* and *S. warneri*) may be predictive of treatment response; and 3) decreases in *S. aureus* and *S. lugdunensis* population size are associated with treatment response (10). However, the relationship between phototherapy and the gut microbiome – which comprises the most microbial-rich niche in the body and carries important roles in cancer immunity and defense against pathogens (20) – has yet to be studied in CTCL. We herein investigated the relationship between the gut microbiome and nbUVB treatment in CTCL. This study attempts to both elucidate the impact of nbUVB on the CTCL gut microbiome and to provide a framework for evaluating the biological relevance of gut microbial differences distinguishing patients and healthy individuals.

## Materials and methods

### Participants

Ethical approval was obtained from the Northwestern University Institutional Review Board (STU00209226). Patients were consented and enrolled at the Northwestern University Cutaneous Lymphoma specialty clinic between 2019 and 2021 in compliance with the Declaration of Helsinki. Personal data and stool samples were collected from 21 patients with clinically and biopsy-confirmed CTCL, as reviewed by an expert dermatopathologist (JG).

Thirteen patients were prescribed a treatment regimen of nbUVB phototherapy, and the remaining 8 participants used non-nbUVB standard of care treatments. In addition to nbUVB treatment, 19 patients were utilizing topical therapy, of which 63% (n=12) were utilizing corticosteroid monotherapy. Seven patients utilized systemic treatments, and 2 patients were treatment naïve. Patients were surveyed on their diet and concurrent medications. Patients who utilized antibiotics within the 4 weeks prior to collection were excluded. Modified Severity-Weighted Assessment Tool (mSWAT) was assessed by the principal investigator (XAZ). The healthy control group (HC) was comprised of 13 patients without CTCL or other active skin disease. Statistical analyses were performed utilizing GraphPad Prism v9.5.1.

### Sample collection and DNA extraction

Patients were provided with sterile swabs and tubes, and were instructed to swab toilet paper after defecation. Samples were then sent by overnight mail to our facility and stored at -80°C (21, 22). Genomic DNA was extracted with Maxwell^®^ RSC Fecal Microbiome DNA kit (Promega; Madison, WI, USA) on a Maxwell^®^ RSC Instrument, following the manufacturer’s protocol.

### 16S rRNA amplification and sequencing

Genomic DNA was prepared for sequencing using a two-stage amplicon sequencing workflow, as described previously (23). Genomic DNA was PCR-amplified using primers targeting the V4 region of microbial 16S rRNA genes. The primers, 515F modified and 806R modified, contained 5′ linker sequences compatible with Access Array primers for Illumina sequencers (Fluidigm; South San Francisco, CA) (24). PCRs were performed in a total volume of 10 μL using MyTaq™ HS 2X Mix (Bioline), primers at 500 nmol/L concentration, and approximately 1000 copies per reaction of a synthetic double-stranded DNA template (described below). Thermocycling conditions were 95°C for 5′ (initial denaturation), followed by 28 cycles of 95°C for 30 s, 55°C for 45 s, and 72°C for 30 s. The second-stage PCR reaction contained 1 μL of PCR product from each reaction and a unique primer pair of Access Array primers. Thermocycling conditions were 95°C for 5′ (initial denaturation), followed by 8 cycles of 95°C for 30 s, 60°C for 30 s, and 72°C for 30 s. Libraries were pooled and sequenced on an Illumina MiniSeq sequencer (Illumina; San Diego, CA, USA) with 15% phiX spike-in and paired-end 2 × 153 base sequencing reads.

### Basic processing

Sequencing resulted in a total of 4,827,112 reads with an average of 69,958 reads per sample. Forward (F) and reverse (R) reads were trimmed using cutadapt v3.5 to remove primer sequences (25). All reads were trimmed and filtered based on quality using the default parameters within dada2 v1.22 (26) with the following parameters: the truncLen setting was disabled, and maxEE for all reads was set to 2,2. The error models were initiated using 5x10^8^ randomized bases for the F and R sets. F and R amplicon sequencing variant (ASV) read pairs were merged with a maxMismatch of 0 and a minOverlap of 6 bases following subsequent denoising with dada2’s divisive partitioning machine learning approach. Merged pairs with lengths outside the 252-255 base window were eliminated. Chimeric sequences were identified and removed using a *de novo* approach within dada2 assessing the entire sequence pool as a whole. ASVs were assigned taxonomy using DECIPHER v2.22 with the SILVA v138 training set (27, 28). Suspected contaminant ASVs above the default prevalence threshold of 0.1 were classified as potential environmental artifacts and subsequently removed from the dataset. To further refine the data set, an abundance filter of 0.1% and a prevalence filter of 10% were applied. Following QC processing, decontamination, and filtering, there were a total of 2,655,033 merged read pairs with an average of 38,478 per sample. Only samples with minimum 1000 reads following processing were retained. Patient samples were paired across time (pre-nbUVB, post-nbUVB) and only complete patient-matched sets were retained for analysis.

### Functional analysis

The output from the 16S rRNA amplicon denoising was used as input for the PICRUSt2 predicted metagenome generation pipeline (29) The seqtab.nochim file output from dada2 denoising of the 16S rRNA gene amplicon sequencing, along with the associated metadata table, were used to generate a fasta file containing the ASV sequences. The abundance table as well as the generated fasta file were used with default PICRUSt2 v2.5.2 (30) parameters to generate a predicted metagenome. The output of this approach generated predicted abundances of Enzyme Commission (EC) and KEGG pathway abundances.

### Statistical analysis

The samples in the cleaned ASV table were visually evaluated using phyloseq v1.38 (31). α-diversity metrics were generated using the ASV table rarefied to 1000 sequences. Differences in α-diversity (Observed OTUs, Chao1, Shannon, and Simpson indices) between patient sets were calculated using Wilcoxon rank-sum paired tests from the stats R package (32). β-diversity metrics were generated using the rarefied ASV table. Principal coordinate analysis (PCoA) with Bray-Curtis dissimilarity was performed to identify β-diversity using an ADONIS2 method (permutations = 500) of vegan v2.5.7 (33).

Differential abundance analysis for the denoised 16S rRNA gene amplicon data, as well as the PICRUSt2-predicted metagenome EC and pathway abundances were conducted by MaAsLin2 (34) using the proportional ASV table, with ASVs removed if they had less than 4 counts or a prevalence below 10% across the sample set. Significant ASVs were only considered if they achieved a false discovery rate (FDR)-adjusted p-value of <0.05 (q-value). The predicted EC and pathway abundance data tables were filtered with a count cut-off of 4 units, a prevalence filter of 20%, and a low-variance filter set at 10% using the inter-quantile range. Following this, these data were subjected to a centered log ratio (CLR) transformation to account for predicted metagenome library size variation from sample to sample. These filtered, transformed data tables from the PICRUSt2 pipeline were assessed for differentially abundant predicted ECs and pathways using MaAsLin2 and DeSeq2 (version v2.5.2) (30).

## Results

### Participant characteristics

**Table 1** summarizes the clinical characteristics of CTCL patients (n=21) and HC (n=13). All enrolled subjects were from the same geographical region (Chicago metropolitan area, United States) to reduce environmental variation on the microbiota (35). The median age of CTCL patients was 58.2 years (range 33-76), and 13 patients (62%) were male. The median age of HC was 53.4 years (range 25-79). There were no significant differences in age, sex, race, or Fitzpatrick phototypes between patients and HC (p>0.05). There were no significant changes in diet or systemic treatments between visits (p>0.05). Fifteen patients had mycosis fungoides, 3 had Sezary syndrome, and 3 had other CTCL diagnoses.

**Table 1:** Patient demographic and clinical characteristics.

	nbUVB Treated Patients (T)		Not-Treated (NT)	Healthy Controls (HC)	CTCL vs HC, p-value	R v NR, p-value	NT vs T, p-value
	Responders (R)	Non-Responders (NR)					
**N**	6	7	8	13			
**Mean Age (range), yr**	60.2 (45-76)	55.9 (33-70)	58.8 (47-65)	53.4 (25-79)	0.32	0.60	0.86
Sex (%)							
Male	3 (50.0)	4 (57.1)	6 (75.0)	7 (53.8)	0.73	1.00	0.40
Female	3 (50.0)	3 (42.9)	2 (25.0)	6 (46.2)			
Race (%)							
White	6 (100.0)	4 (57.1)	8 (100.0)	11 (84.6)	1.00	0.19	0.26
Other	0 (0.0)	3 (42.9)	0 (0.0)	2 (15.4)			
FST (%)							
Light (I-III)	6 (100.0)	5 (71.4)	8 (100.0)	12 (92.3)	1.00	0.46	0.50
Dark (IV-VII)	0 (0.0)	2 (28.6)	0 (0.0)	1 (7.7)			
CTCL subtype (%)							
MF	5 (83.3)	5 (71.4)	5 (62.5)			0.23	0.55
SS	0 (0.0)	2 (28.6)	1 (12.5)				
Other	1 (16.7)	0 (0.0)	2 (25.0)				
Non nbUVB treatments (%)							
Skin-directed	4 (80.0)	4 (42.9)	4 (50.0)			1.00	0.38
Systemic and skin-directed	1 (16.7)	2 (42.9)	4 (50.0)				
Mean Change in mSWAT (range)							
pre- versus post-nbUVB	-11.5 (-21 - -1)	9.4 (0-23)	-0.9 (-6 - 3)			0.001	0.13
Comorbidities (%)							
HTN	3 (50.0)	4 (57.1)	4 (50.0)	4 (30.8)	0.30	1.00	1.00
HLD	3 (50.0)	4 (57.1)	4 (50.0)	4 (30.8)	0.30	0.56	1.00
DM	0 (0.0)	3 (42.9)	1 (12.5)	3 (23.1)	1.00	0.19	1.00
GERD	0 (0.0)	2 (28.6)	2 (25.0)	6 (46.2)	0.25	0.46	0.62

CTCL, cutaneous T-cell lymphoma; DM, diabetes mellitus; FST, Fitzpatrick skin phototype; GERD, gastroesophageal reflux disease; HLD, hyperlipidemia; HTN, hypertension; mSWAT, modified Severity Weighted Assessment Tool; nbUVB, narrowband ultraviolet B.

Of the 21 patients with CTCL, 13 underwent nbUVB therapy. Eight sex-, age-, and race-matched patients who were not treated (NT) with nbUVB therapy were also included. Skin disease severity was assessed using mSWAT (36). Of patients receiving phototherapy, 6 were responders (R), defined as a decrease in mSWAT, and 7 were non-responders (NR), defined as no change (n=2) or increase in mSWAT (n=5). There were also no differences in patient characteristics or comorbidities between the R and NR. Additionally, there were no significant differences in clinical or TNMB staging, large cell transformation, or lactate dehydrogenase levels between groups. Median change in mSWAT was -11.5 for responders (range -21 - -1) and 9.4 for non-responders (range 0-23).

### Biodiversity and microbial communities across groups

**Figure 1:**
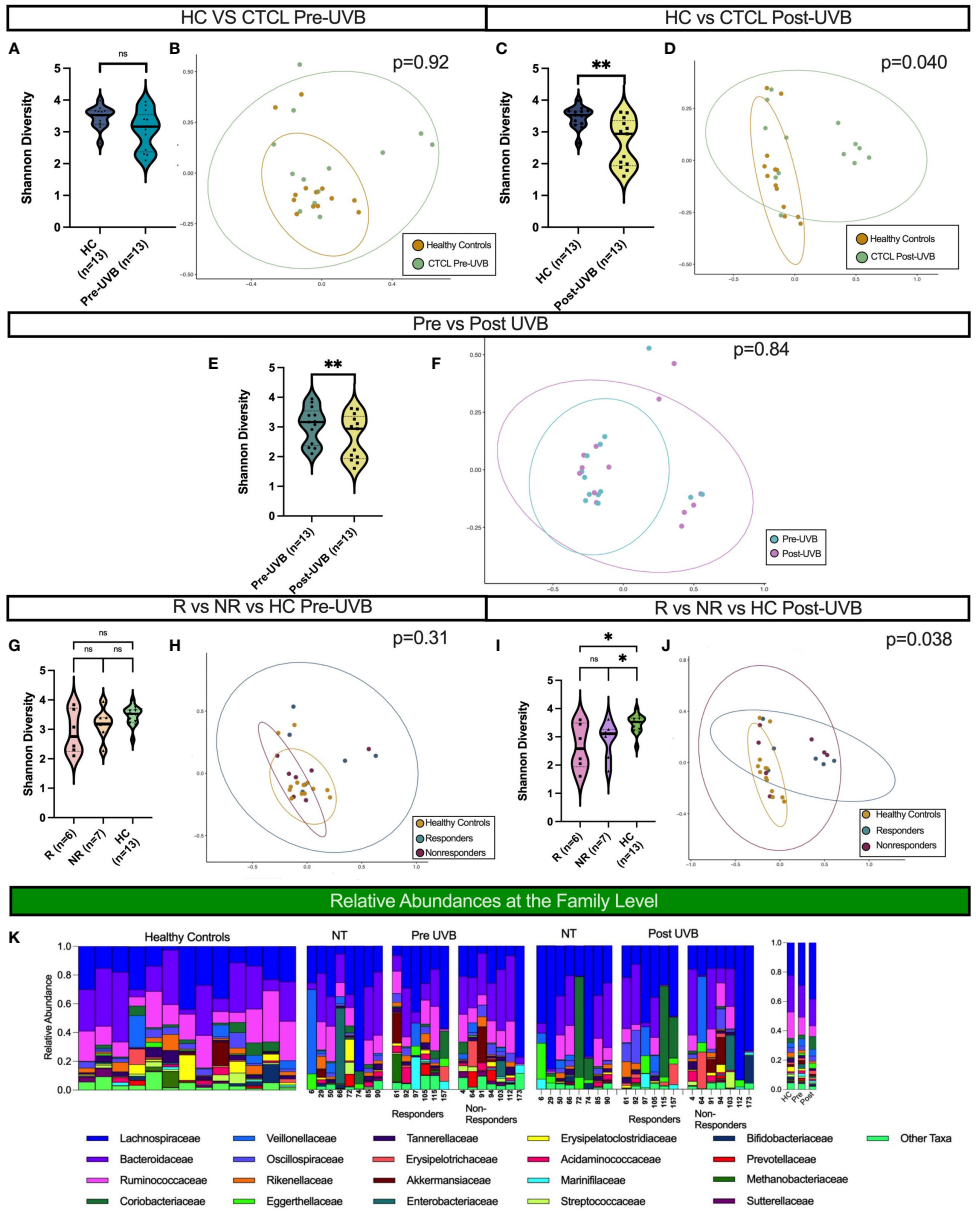
Distinct microbial communities comprise the gut microbiota of CTCL patients following nbUVB treatment, but not before. Black horizontal lines for Shannon diversity violin plots indicate group median. **(A, B)** There were no differences in α- or β-diversity between HC and the 13 CTCL treated patients pre-nbUVB (Shannon p=0.10, Bray-Curtis p=0.92). **(C, D)** Post-nbUVB, the 13 treated CTCL patients had significantly lower α-diversity and significant Bray-Curtis dissimilarity when compared to HC (Shannon p=0.0051, Bray-Curtis p=0.040). **(E)** Treated patients had a significantly lower α diversity post-treatment compared to pre-treatment (Shannon p=0.0024). **(F)** Community structures were not distinct pre- versus post-treatment (Bray-Curtis p=0.84). **(G)** There were no differences in α-diversity between R, NR, and HC pre-treatment (R vs NR, Shannon p = 0.53; NR vs HC, p = 0.24; R vs HC, Shannon p = 0.21). **(H)** There were no differences in community structure between R, NR and HC pre-treatment (Bray-Curtis p = 0.31). **(I)** There were no differences in α-diversity between R and NR post-treatment, but both had differences in α-diversity post-treatment when compared to HC (R vs NR, Shannon p = 1; NR vs HC, p=0.011; R vs HC, p=0.022). **(J)** Community structures of R, NR and HC were different from each other post-treatment (Bray Curtis p = 0.038). **(K)** Relative abundance of bacterial taxa in stool samples at the family level. Relative abundances were categorized into healthy controls, non-treated CTCL patients, nbUVB responders, and nbUVB non-responders. The mean relative abundances for HC, CTCL pre-nbUVB, and CTCL post-nbUVB are shown on the right. ns, not significant, P > 0.05; *: P ≤ 0.05; **: P ≤ 0.01.

Consistent with our prior gut microbiome results, we did not observe a significant difference in phylogenetic diversity or microbial community structures when examining our nbUVB-treated CTCL cohort in comparison to age-matched HC (Shannon p=0.10, Bray-Curtis p = 0.92) (**Figures 1A, B**) (11). However, phylogenetic diversity was significantly lower in CTCL patients with more severe disease (mSWAT >15) compared to HC (p=0.018), as demonstrated in our previous report (11).

Treatment with nbUVB decreased microbial diversity and altered the community structure of the CTCL gut microbiome in comparison to HC. While there was no significant difference in α-diversity between CTCL patients and HC at the initial visit, treated CTCL patients had significantly decreased α-diversity and a distinct gut microbial signature post-nbUVB when compared to HC (Shannon p=0.0051, Bray-Curtis p=0.040) (**Figures 1C, D**). The 13 nbUVB-treated CTCL patients also had lower phylogenetic diversity post-treatment when compared to pre-treatment (Shannon p=0.0024), though β-diversity was not significantly different (**Figures 1E, F**).

No differences in phylogenetic diversity or microbial composition were identified between R and NR, before or after treatment (**Figures 1G–J**). Nevertheless, both R and NR exhibited decreased phylogenetic diversity compared to HC post-treatment (p=0.022 and p=0.011, respectively) (**Figure 1I**).

The most abundant bacterial taxa at the family level from all samples are presented in **Figure 1K**. Overall, *Lachnospiraceae* was the most abundant family present in CTCL patients, both before and after nbUVB treatment in treated patients and in NT patients, and the second most abundant taxa in HC. *Bacteroidaceae* was the most abundant family in HC and the second most abundant in CTCL patients. *Ruminococcaceae* was the third most abundant taxa in all participants included in our study.

### Taxonomic differences between CTCL patients and healthy controls

**Figure 2:**
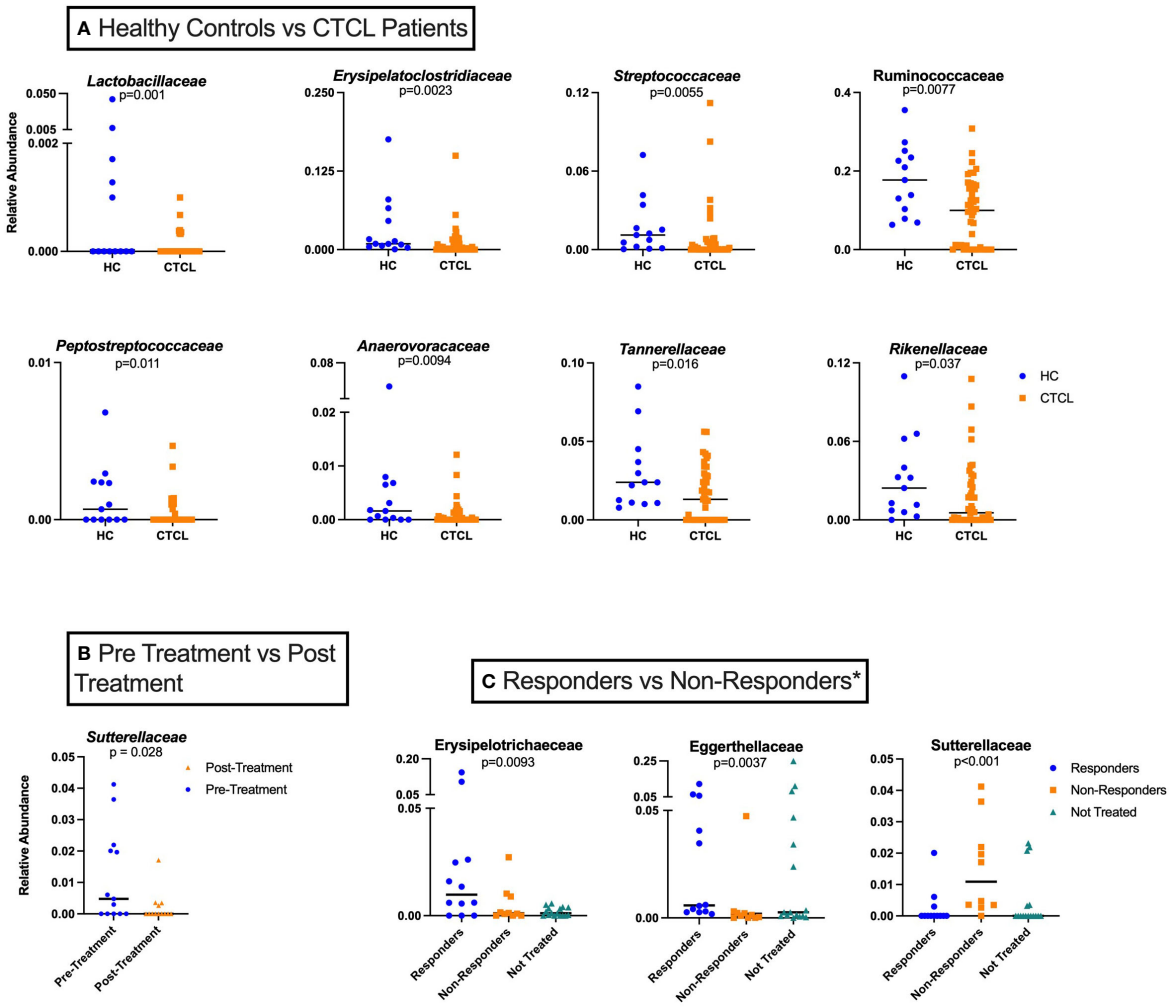
Taxon-by-taxon analysis amongst various study groups analyzed. **(A)** Individual taxa differences between HC (n=26) and CTCL patients, both treated and untreated (n=42). **(B)** Relative abundance of *Sutterellaceae* for all treated CTCL patients prior to nbUVB differed from that of patients following nbUVB (n=13). **(C)** Differences in individual taxa pre- and post-nbUVB between responders (R) (n=12), non-responders (NR) (n=10), and not treated (NT) CTCL patients (n=16)*. In this analysis, NR are defined only as those who had an increase in their mSWAT score.

We first performed taxon-by-taxon analysis comparing all CTCL patients (NT, pre-nbUVB, and post-nbUVB) and HC. This analysis revealed that *Lactobacillaceae* and *Erysipelatoclostridiaceae* were significantly higher in HC than in CTCL patients (p=0.001, q=0.045 and p=0.002, q=0.049). Other taxa that trended higher in HC samples included *Ruminococcaceae, Streptococcaceae, Tannerellaceae, Anaerovoracaceae, Peptostreptococcaceae, and Rikenellaceae* (**Figure 2A**). These data are consistent with our prior findings that demonstrated the loss of healthy commensal bacteria, including *Lactobacillaceae*, in the gut microbiome of CTCL patients (11, 37). No other specific taxa were significantly higher (p<0.05, q<0.05) or trended higher (p<0.05, q>0.05) in CTCL patients compared to HC.

**Figure 3:**
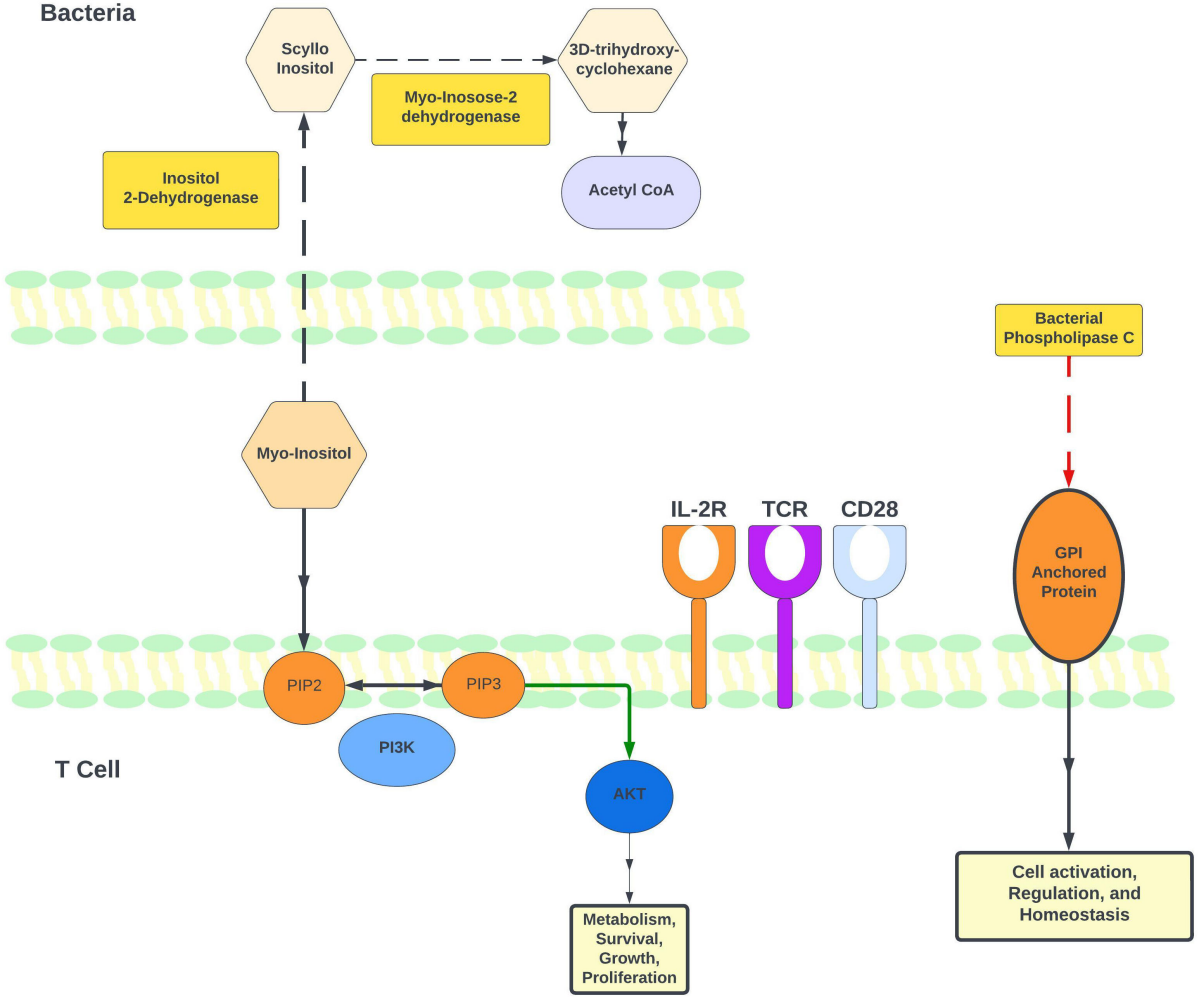
Regulation of myo-inositol levels and its role in T-cell immune modulation. Based on available literature and our PICRUSt2 analysis, this figure illustrates the proposed model for how myo-inositol and its degradation pathway may be involved in immune regulation. Our gut microbiome results indicate higher levels of P562-PWY (myo-inositol degradation I) in CTCL patients undergoing phototherapy. Notably, 2 enzymes in this pathway were identified as higher in CTCL patients: EC 1.11.18 (inositol 2-dehydrogenase) and EC 4.2.1.44 (myo-inosose-2 dehydratase). Additionally, EC 3.1.4.3 (phospholipase C [PLC]) is lower in patients after nbUVB treatment. Bacterial PLC and is known to promote T-cell activation through its actions on glycosylphosphatidylinositol (GPI) anchors.

We performed PICRUSt2 analysis on genomic results from 16S rRNA amplification and sequencing to determine differences in individual enzyme and enzymatic pathway abundances between comparison groups. Enzymes and pathways related to inositol regulation, which plays an important role in both T-cell effector and regulatory functions (38), were repeatedly higher in CTCL patients when compared to HC (**Figure 3**). In comparison to HC, CTCL patients had increased relative abundance of sequences that map to enzymes EC:1.1.1.370 (scyllo-inositol 2-dehydrogenase), EC:4.2.1.44 (myo-inosose-2 dehydratase), EC:5.3.99.11 (2-keto-myo-inositol isomerase), EC:1.1.1.18 (inositol 2-dehydrogenase), and EC:1.1.1.369 (D-chiro-inositol 1-dehydrogenase). When examining pathways that were differentially abundant, P562-PWY (myoinositol degradation pathway) and PWY-7237 (myo-, chiro- and scyllo-inositol degradation) were higher in CTCL patients.

### Changes in taxonomic relative abundances with nbUVB treatment

Next, we performed taxon-by-taxon analysis comparing patients before and after receiving nbUVB, regardless of their treatment response. This analysis revealed that the relative abundance of *Sutterellaceae* trended lower in CTCL patients after receiving nbUVB (p=0.028, q=0.67) (**Figure 2B**). *Sutterellaceae* has been previously shown to be pro-inflammatory by degrading IgA, which helps provide protective immunity for intestinal epithelium (39).

PICRUSt2 analysis was performed to analyze differences in enzyme and pathway abundances between nbUVB-treated CTCL patients, NT CTCL patients, and HC. EC:1.1.1.370 (scyllo-inositol 2-dehydrogenase (NAD(+)) was significantly higher in patients receiving phototherapy treatment, and P562-PWY (myoinositol degradation pathway) was significantly higher in this group as well. By mapping pathways to individual taxa, we were able to determine which bacterial taxa contributed to certain enzymes and pathways. *Lachnospiraceae*, a robust butyrate producer, was the most frequent contributor to EC:1.1.1.370 (scyllo-inositol 2-dehydrogenase) and pathway P562-PWY (myo-inositol degradation pathway). PICRUSt2 analysis on CTCL patients pre- and post-nbUVB treatment revealed a lower post-treatment abundance of EC:3.1.4.3 (bacterial phospholipase C), which hydrolyzes phosphatidylinositol and can cleave glycosylphosphatidylinositol (GPI)-anchored proteins (**Figure 3**).

### Responders vs. non-responders

Finally, we examined whether specific gut bacterial taxa were associated with disease response or non-response to nbUVB treatment (**Figure 2C**). When examining responders against only patients who worsened with treatment (excluding stable patients), we found that several taxa diverge between the two groups. These taxa included *Sutterellaceae*, which was significantly higher in those who worsened with nbUVB compared to those who improved or were not treated (p<0.001) (**Figure 2C**). Additionally, the relative abundances of *Eggerthellaceae* and *Erysipelotrichaceae* trended higher in R (p=0.0037 and 0.0093, respectively), and that of *Akkermansiaceae* trended higher in NR (p=0.026). Notably, post-nbUVB, *Lachnospiraceae* trended higher in those who were R versus NR (p=0.010).

## Discussion

Utilizing 16S rRNA gene amplicon sequencing and PICRUSt2 predictive metagenomics, we elucidated the gut microbial profiles of 21 CTCL patients and 13 healthy, age-matched individuals. To our knowledge, this is the first study to longitudinally characterize the gut microbiome of CTCL patients treated with skin-directed nbUVB. Our prior cross-sectional analysis evaluating the gut microbiota of CTCL patients found more severe disease is associated with reduced microbial diversity (11). We also previously studied the effect of nbUVB on the skin microbiome in CTCL but did not examine the gut microbiome (10). Having previously established that CTCL is associated with altered gut microbiota and that nbUVB response is coupled with changes in the CTCL skin microbiome, in this present study we sought to explain the relationship between the CTCL gut microbiome and nbUVB phototherapy. Limited data suggest nbUVB can impact the gut microbiome (40, 41), but this has yet to be explored in CTCL. Importantly, our current longitudinal dataset allowed us to examine whether changes to the gut microbiome are indicative of treatment response to skin-directed nbUVB. Moreover, this analysis included 26 new specimens from 5 additional CTCL patients and second collection time points, enabling us to explore this unique research question with a larger sample size and greater statistical power.

This work further contributes to a growing body of knowledge that suggests CTCL is a disease associated with global dysbiosis (10–16). The data herein support our previous findings on the CTCL gut microbiome, namely that patients with more advanced disease demonstrated decreased phylogenic diversity and loss of beneficial commensal taxa in comparison to healthy controls (11). We again demonstrated reduced relative abundance of commensal bacteria such as *Lactobacillaceae* in CTCL patient samples compared to age-matched healthy controls. Surprisingly, we found that bacterial phylogenetic diversity decreased with nbUVB treatment regardless of response status. While α-diversity did not differ between HC and CTCL patients prior to nbUVB, it was significantly lower amongst post-nbUVB CTCL patients compared to both HC and pre-nbUVB CTCL samples. These observations suggest that nbUVB treatment had the greatest impact on α-diversity. Interestingly, this finding contrasts with our data on the impact of nbUVB on the skin microbiome in CTCL, in which α-diversity increased in nbUVB responders but not in non-responders (10). It has also been proposed that UVB may induce changes in local and adaptive immune cells, which then traffic to the gut to shape the intestinal microbiome (40). Phototherapy may prime systemic immunity in a manner that leads to the decreased microbial diversity in nbUVB-treated CTCL patients. Nevertheless, α-diversity is one of many constantly-evolving metrics to describe a microbiome community structure (42). This change should also be noted in the context of other descriptive measures, such as the reduction in *Sutterellaceae*, as described below.

Very few prior studies have examined the response of the gut microbiome to nbUVB phototherapy. One group examined the effects of nbUVB over one week (3 sessions) on the gut microbiome of healthy individuals and found that phylogenetic diversity increased in participants who did not receive vitamin D supplementation. Some healthy commensal taxa such as *Lachnospiraceae* increased after nbUVB (40). Within our cohort, there were no differences in α-diversity before and after nbUVB treatment between patients who took vitamin D and those who did not (p=0.92 and p=0.77, respectively), but we did note a trend towards increased *Lachnospiraceae* relative abundance in nbUVB responders regardless of vitamin D status. Rungjang et al. investigated how gut microbiota change after nbUVB therapy in psoriasis patients and identified increased levels of *Lactobacillaceae* in responders (41). While we did not identify this shift in nbUVB responders, we did find that *Lactobacillaceae* was significantly more abundant in healthy controls compared to CTCL patients.

We found that *Sutterellaceae* decreased with nbUVB treatment regardless of response and increased *Sutterellaceae* abundance was associated with non-responders. Notably, *Sutterella* has been found to be increased in the gut microbiome of children with atopic dermatitis, another chronic inflammatory skin disorder with a Th2 immune shift. This association further supports a connection between immunological profiles and gut microbial communities (11, 43). Moreover, *Sutterellaceae* has previously been linked to ulcerative colitis and can degrade IgA, potentially reducing gut epithelial immunity against bacterial infiltration (39). Thus, *Sutterellaceae* may contribute to a pro-inflammatory environment in CTCL.

Recent reports have focused on the gut microenvironment and the metabolized products of gut microbiota, and their effect on T-cell regulation in chronic inflammatory skin diseases (44, 45). In our study, PICRUSt2 predictive analyses revealed that shifts in specific microbial sequences may contribute to increased myo-inositol degradation in CTCL patients who received phototherapy. Myo-inositol is used to build phosphoinositides, which are subsequently metabolized to form second messengers with important roles in T-cell development and function (38). Many of these second messengers are acted on by PI3Ks, lipid kinases that play a central role in TCR signaling and the balance between immune regulation and activation of various effector CD4+ T-cells (46, 47). Inhibition of the PI3K pathway is notably being investigated for treatment of CTCL (48). Our analysis further revealed *Lachnospiraceae* contributed most frequently to the myo-inositol degradation pathway. As the PICRUSt2 data was generated by differential bacterial abundances between the treatment and non-treatment groups, these findings reflect the functional implication that bacteria, including species from the *Lachnospiraceae* family, may regulate myo-inositol degradation following nbUVB and thereby indirectly contribute to modulation of the PI3K pathway. Because *Lachnospiraceae* relative abundance trended higher in nbUVB responders than in non-responders, increased myo-inositol degradation and PI3K pathway modulation may, in part, explain successful treatment response.

PICRUSt2 analysis also predicted a decrease in bacterial phospholipase C (PLC) in patients after treatment with nbUVB. Previous research has demonstrated the ability of bacterial PLC to modulate the host cell immunity by degrading glycosyl phosphatidyl inositol (GPI), which is only expressed in eukaryotic cells (49). Furthermore, GPI anchored cell surface proteins are important for T-cell activation (50, 51). This finding suggests that a decrease in bacterial PLC following nbUVB may potentially dampen malignant T-cell activity. **Figure 3** illustrates a proposed mechanism for myo-inositol and bacterial PLC’s impact on T-cell activity.

It is important to also address changes in gut β-diversity following nbUVB treatment. While there were no differences between CTCL and HC before treatment, microbial communities did differ after treatment. Given this finding, one can postulate that phototherapy may not return patients to a “healthy” baseline microbial community, but rather the community becomes entirely distinct from that of healthy controls.

When examining the relative abundance of specific taxa in responders from non-responders, we observed that both *Eggerthellaceae* and *Erysipelotrichaceae* were higher in responders than non-responders. *Eggerthellaceae* is known to degrade polyphenols in the gut and has anti-inflammatory properties, both of which may play a role in nbUVB response (39, 52). Furthermore, *Erysipelotrichaceae* is a common member of a healthy gut microbiota, and has been noted to be highly coated by IgA, a protective immunoglobulin, relative to other gut bacteria (53). When analyzing only post-nbUVB results, we also observed *Lachnospiraceae* relative abundance trended higher in responders compared to non-responders. *Lachnospiraceae* produces the short chain fatty acid butyrate that functions as a potent histone deacetylase inhibitor – the mechanism of action of 2 FDA-approved CTCL treatments, vorinostat and romidepsin (54–56).

Limitations of this study include small sample size, which limits the statistical power of this study. However, it is important to note that CTCL is a relatively rare disease, which limits the number of patients available for study at large centers such as ours. To reduce the presence of confounders, our study participants were extensively characterized and rigorously controlled. Moreover, this study was designed to be longitudinal to control for inter-individual variation. Finally, while we controlled for patient diet and medications to the best of our ability, these factors still have the potential to influence microbial results. Our study excluded patients who utilized antibiotics within the prior month, but it did not exclude patients who may have had a more remote history of antibiotic usage. We chose patients who were naïve to or had a long and consistent history of the same systemic CTCL therapy (6+ months) to minimize the impact of alternative treatments on the gut microbiome. Lastly, there were no significant differences in comorbidity profiles amongst study groups, thus minimizing differences in non-CTCL medication use.

This study represents the first analyses of gut microbial changes in patients with CTCL following nbUVB treatment. Our findings concur with our previous work, which identified decreased phylogenic diversity in patients with advanced CTCL. We expanded upon these prior findings by identifying differences in phylogenetic and microbial diversity amongst patients following nbUVB therapy, as well as changes in specific taxa abundances after nbUVB. Future directions include functionally validating alterations in the inositol pathway through experimental models, investigating the influence of *Lachnospiraceae* on butyrate levels in CTCL, and analyzing the gut microbiome at the strain level through shotgun metagenomic sequencing. Additional studies are needed to further clarify the relationship between nbUVB, vitamin D, and the gut microbiome in larger cohorts (41). Our work provides new insight into the intimate connection between CTCL and gut microbiota. Continued study of the relationship between the gut microbiome and the key enzymes and metabolic byproducts within it may allow novel therapeutic options for CTCL to be uncovered.

## Data availability statement

The data presented in the study are deposited in the NCBI Short Read Archive, accession number PRJNA1060341 (https://www.ncbi.nlm.nih.gov/bioproject/1060341).

## Ethics statement

The studies involving humans were approved by Northwestern University Institutional Review Board. The studies were conducted in accordance with the local legislation and institutional requirements. The participants provided their written informed consent to participate in this study.

## Author contributions

WN: Writing – original draft, Writing – review & editing. LC: Writing – original draft, Writing – review & editing. GE: Data curation, Formal analysis, Methodology, Software, Writing – original draft, Writing – review & editing. MH: Conceptualization, Data curation, Investigation, Writing – review & editing. TG: Data curation, Investigation, Writing – review & editing. SG: Writing – review & editing. PS: Writing – review & editing. JG: Conceptualization, Investigation, Writing – review & editing. MB: Data curation, Formal analysis, Methodology, Software, Writing – original draft, Writing – review & editing. XZ: Conceptualization, Funding acquisition, Investigation, Methodology, Project administration, Writing – original draft, Writing – review & editing. MA: Formal analysis, Software, Writing – review & editing. SR: Formal analysis, Software, Writing – review & editing.
