# Supplementary material for: Gut microbiota analyses of cutaneous T-cell lymphoma patients undergoing narrowband ultraviolet B therapy reveal alterations associated with disease treatment
Source: Front Immunol. 2024 Jan 11;14:1280205. doi: 10.3389/fimmu.2023.1280205 (PMC10808320; doi:10.3389/fimmu.2023.1280205)
Supplement: Supplementary file 1 [file DataSheet_1.docx]

| **Sex** | **Age** | **Race** | **FST** | **CTCL Subtype** | **Stage** | **mSWAT change** | **Other Treatments** | **Non-CTCL medications** |
| --- | --- | --- | --- | --- | --- | --- | --- | --- |
| ***Responders*** | | | | | | | | |
| M | 49 | White | II | MF | IIB | -7.5 | TCS, Acitretin | Pentoxifylline |
| M | 61 | White | I | PCAETCL | IIA | -19.5 | TCS | Aspirin, Cholecalciferol, Ramipril, Zolpidem |
| F | 69 | White | II | MF | IA | -9 | TCS | Amlodipine |
| M | 61 | White | I | MF | IB | -21 | TCS | Atorvastatin, Fenofibrate, Losartan |
| F | 45 | White | III | CD8+ MF | IA | -11 | None | None |
| F | 76 | White | II | MF | IA | -1 | TCS | None |
| ***Non-Responders*** | | | | | | | | |
| M | 33 | White | III | FMF | IB | 0 | TCS | Metformin, Multivitamin, Rosuvastatin |
| F | 35 | Asian | III | MF | IB | 0 | None | None |
| M | 69 | White | II | FMF | IB | 4 | TCS, Acitretin | Amlodipine, Simvastatin, Valsartan-HCTZ |
| M | 66 | Black | V | SS | IV | 22 | TCS | Amlodipine, Aspirin, Doxepin, Escitalopram, Lisinopril, Metoprolol |
| M | 70 | White | II | MF | IA | 10 | TCS, Bexarotene, Methotrexate | Alprazolam, Amlodipine, Aspirin Gabapentin |
| F | 52 | Black | V | FMF | IIB | 23 | TCS | Amlodipine |
| F | 66 | White | II | SS | IV | 7 | TCS | Gabapentin, Ketoconazole, Prednisone, Zolpidem |
| ***Not Treated*** | | | | | | | | |
| F | 55 | White | I | SS | IV | -6 | TCS | Alprazolam, Atorvastatin, Cholecalciferol, Fexofenadine, Levothyroxine, Sertraline |
| M | 63 | White | II | MF | IB | -2 | TCS, Methotrexate | Cetirizine |
| M | 47 | White | II | MF | IB | 0 | TCS | Omeprazole |
| M | 65 | White | II | MF | IA | -1 | TCS | Lisinopril-HCTZ |
| F | 60 | White | II | CTCL NOS/PTCL | IB | -2 | TCS, Bexarotene | Famotidine |
| M | 62 | White | I | CTCL NOS |  | -1.5 | TCS, Bexarotene, Peginterferon alfa-2a | Enalapril, Levothyroxine, Metronidazole, Simvastatin |
| M | 63 | White | II | MF | IB | 3 | TCS, Acitretin, Peginterferon alfa-2a | Amlodipine, Atorvastatin, HCTZ |
| M | 55 | White | II | MF | IIB | 2.5 | TCS | Cetirizine |

**Supplemental Figure 1.** CTCL: cutaneous T-cell lymphoma, F: female, FMF: folliculotropic mycosis fungoides, FST: Fitzpatrick skin phototype, HCTZ: hydrochlorothiazide, M: male, MF: mycosis fungoides, mSWAT: Modified Severity-Weighted Assessment Tool, NOS: not otherwise specified, PCAETCL: primary cutaneous aggressive epidermotropic T-cell lymphoma, TCS: topical corticosteroids


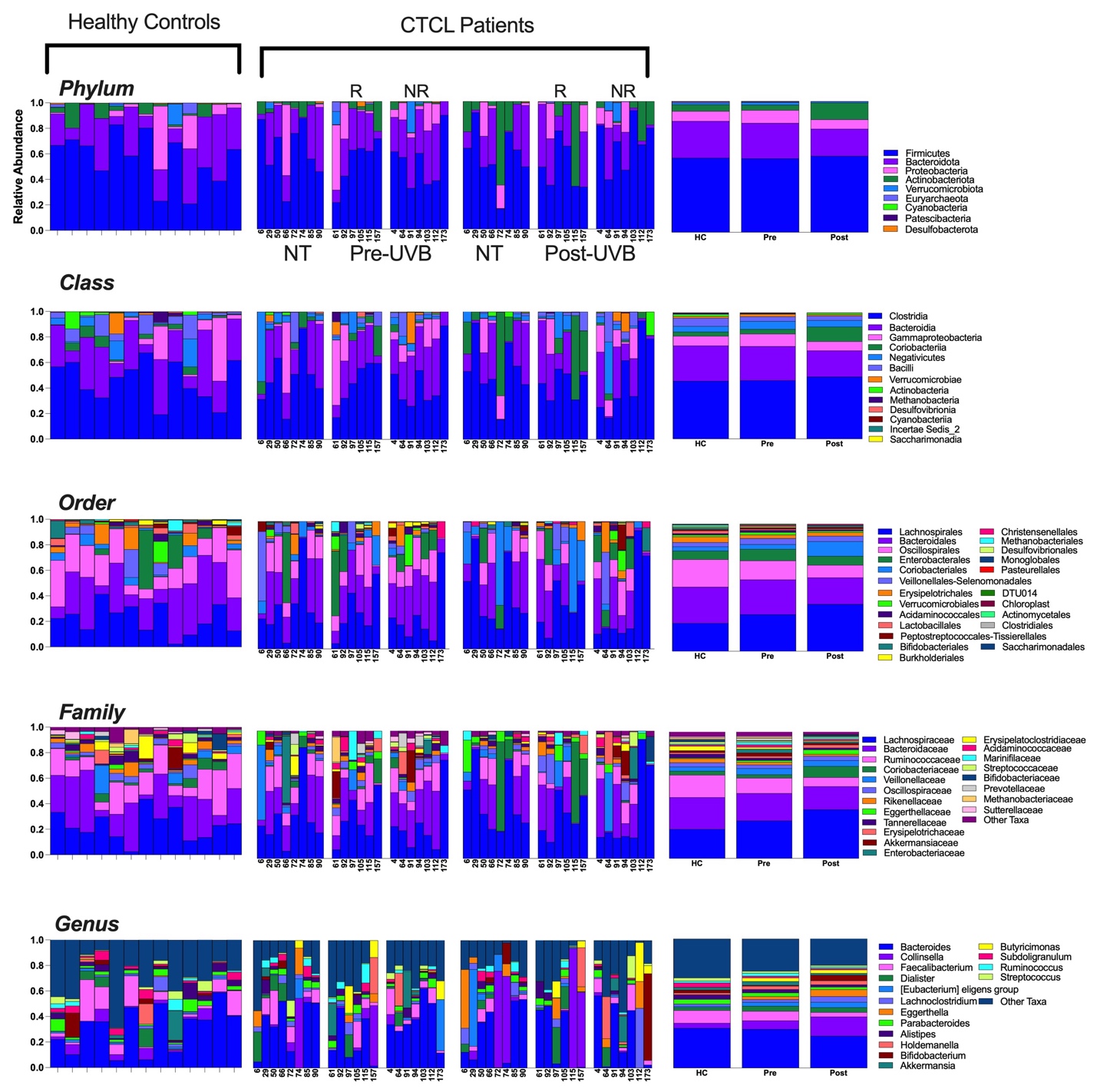


**Supplemental Figure 2.** Breakdown of taxa by Phylum, Class, Order, Family, and Genus, amongst healthy controls (HC), non-treated patients (NT), nbUVB responders (R), and nbUVB non-responders (NR). The mean relative abundances for HC, CTCL pre-UVB (pre), and CTCL post-UVB (post) are shown on the right.
